# A Case Report: Retroperitoneal dedifferentiated liposarcoma with heterologous myogenic differentiation and testicular metastasis

**DOI:** 10.3389/fonc.2026.1836140

**Published:** 2026-06-22

**Authors:** Haosong Ma, Runlin Feng, Guorun Zi, Yiqing Li, Quanlv Xu, Changxing Ke

**Affiliations:** Department of Urology, The Second Affiliated Hospital of Kunming Medical University, Kunming, China

**Keywords:** CDK4 gene, dedifferentiated liposarcoma, heterologous differentiation, MDM2 gene, retroperitoneal tumor, testicular tumor

## Abstract

Dedifferentiated liposarcoma (DDLPS) is the most common retroperitoneal soft tissue sarcoma, characterized by *MDM2* and *CDK4* gene co-amplification. However, DDLPS with heterologous myogenic differentiation is extremely rare and poses a significant diagnostic challenge due to its expression of myogenic markers such as desmin, myogenin, and MyoD1, which can easily lead to misclassification as primary rhabdomyosarcoma. Testicular metastasis of DDLPS is even rarer, with only isolated case reports documented in the literature. We report a case of a 46-year-old male who presented with a large retroperitoneal mass without testicular involvement on initial imaging. He underwent en bloc resection of the retroperitoneal tumor including right nephrectomy and right hemicolectomy. Ten days postoperatively, he developed a right scrotal mass, prompting radical orchiectomy. Initial pathological evaluation suggested rhabdomyosarcoma based on desmin and MyoD1 positivity. However, subsequent expert pathological consultation with fluorescence *in situ* hybridization (FISH) analysis revealed *MDM2* and *CDK4* gene amplification in both specimens, establishing the final diagnosis of DDLPS with prominent heterologous myogenic differentiation. Postoperative chest CT demonstrated early pulmonary metastases, underscoring the aggressive nature of this tumor. This case highlights the critical importance of *MDM2*/*CDK4* FISH testing in accurately diagnosing DDLPS with heterologous differentiation, particularly when myogenic markers are expressed, to avoid misdiagnosis and guide appropriate management.

## Introduction

Dedifferentiated liposarcoma (DDLPS) is the most common retroperitoneal soft tissue sarcoma, accounting for approximately 30-40% of retroperitoneal sarcomas. Its diagnosis relies on the characteristic *MDM2* and *CDK4* gene co-amplification, detectable by fluorescence *in situ* hybridization (FISH) (1). However, DDLPS with heterologous myogenic differentiation (rhabdomyoblastic differentiation) is extremely rare. These tumors express myogenic markers such as desmin, myogenin, and MyoD1, creating a significant diagnostic pitfall that can lead to misclassification as primary rhabdomyosarcoma (RMS) (2, 3). When such tumors involve both the retroperitoneum and testis, the diagnostic challenge is further compounded. Here we report a case of retroperitoneal DDLPS with heterologous myogenic differentiation and subsequent testicular metastasis, highlighting the diagnostic complexities and the critical role of molecular testing.

## Case presentation

**Figure 1:**
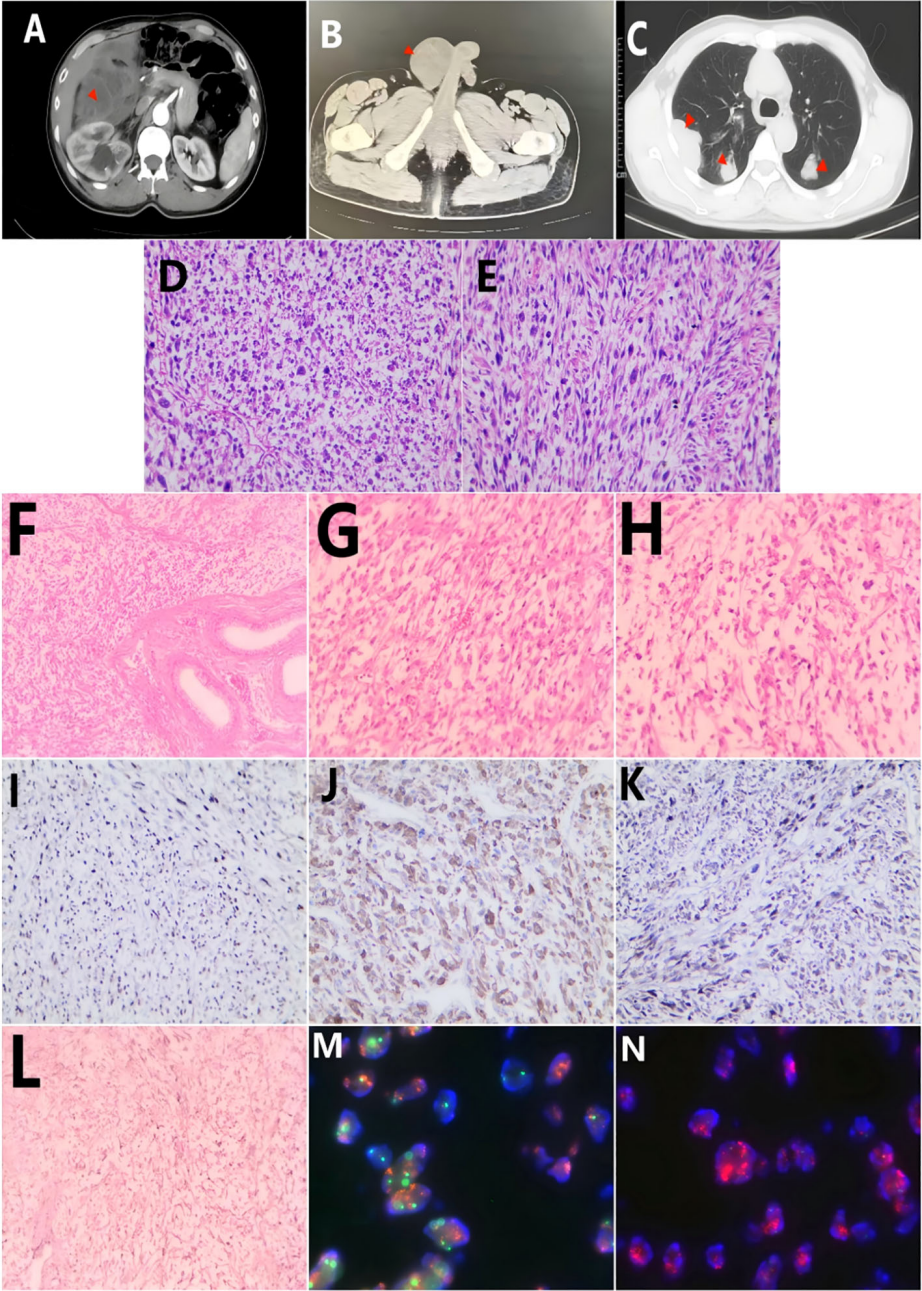
**(A)**: Enhanced abdominal CT scan: Multiple irregular cystic-solid masses in the right mid-abdomen. The upper part of the mass is indistinctly demarcated from the lower pole of the right kidney. Small patchy slightly high-density shadows are visible within some masses. After enhancement, the edges of the masses and the septa within the lesions show significant enhancement, while the high-density shadows within the lesions do not enhance. **(B)**: Enhanced lower abdominal CT scan: A space-occupying lesion in the right scrotum. The edges of the lesion show significant enhancement, while the patchy low-density shadows within the lesion do not enhance. **(C)**: CT imaging reveals multiple irregular solid nodules in both lungs; considering the patient's medical history, metastatic lesions are suspected. **(D)**: HE staining of retroperitoneal mass under low magnification. **(E)**: HE staining of retroperitoneal tumor under high-power microscope. **(F)**: HE staining of testicular masses under low magnification. **(G)**: HE staining of testicular masses under medium magnification. **(H)**: HE staining of testicular masses under high magnification. **(I)**: Immunohistochemistry of the retroperitoneal mass showed Myo-D1 (+). **(J)**: Immunohistochemistry of the retroperitoneal mass showed SMA (+). **(K)**: Immunohistochemistry of the retroperitoneal mass showed Desmin (+). **(L)**: Immunohistochemistry of testicular mass shows Desmin (+). **(M)**: FISH test results: CDK4 gene amplification was detected. **(N)**: Detection results: MDM2 gene amplification was detected.

A 46-year-old male farmer from Yunnan Province, China, presented with a one-month history of a palpable right abdominal mass associated with intermittent dull pain. He had no prior medical history of hypertension, diabetes, chronic kidney disease, or malignancies, and he was not on any long-term medications. He denied smoking, alcohol use, or occupational toxin exposure. His family history was unremarkable for sarcomas or other soft tissue tumors. On admission, his vital signs were as follows: temperature 36.6 °C, pulse 78 beats per minute, blood pressure 153/103 mmHg, and oxygen saturation 99%. His height was 168 cm and body weight was 60 kg (body mass index 21.3 kg/m²). Physical examination revealed a firm, poorly mobile mass measuring approximately 14 cm × 10 cm in the right mid-abdomen without tenderness. The abdomen was soft with no rebound tenderness or guarding, and the scrotum was unremarkable on initial examination. The patient reported no weight loss, fever, or night sweats. Preoperative laboratory workup, including complete blood count, liver function, and renal function, revealed no significant abnormalities. Contrast-enhanced abdominal computed tomography (CT) demonstrated a large, multiseptated, heterogeneously enhancing retroperitoneal mass with a maximum diameter of 15.0 cm, causing right hydronephrosis due to ureteral compression; no testicular abnormality was identified (**Figure 1A**). One week after admission, the patient underwent en bloc resection of the retroperitoneal tumor under general anesthesia. Intraoperatively, the tumor was found to densely adhere to the right kidney and ascending colon, necessitating concomitant right nephrectomy and right hemicolectomy to achieve complete macroscopic resection. The right renal pedicle was doubly ligated and transected, and an end-to-side ileocolostomy was performed. The resected specimen measured 23 cm × 16 cm × 13 cm, and estimated blood loss was 100 mL. After surgery, the patient received intravenous antibiotics (cefuroxime 1.5 g twice daily for five days), patient-controlled analgesia for pain control, and enteral nutrition support. He recovered uneventfully with no postoperative complications, was ambulatory on postoperative day 3, and resumed oral intake on postoperative day 4. Pathological examination of the resected specimen revealed a 17.0 cm × 16.0 cm × 13.0 cm high-grade sarcoma with extensive necrosis. Although the original pathology report did not explicitly describe a well-differentiated liposarcoma component, the diagnosis of DDLPS implies its presence at the tumor periphery, a finding commonly observed in such tumors. Hematoxylin and eosin (H&E) staining of the retroperitoneal tumor showed a proliferation of atypical spindle cells with areas of necrosis at low magnification (**Figure 1D**) and pleomorphic nuclei with occasional rhabdoid cells at high magnification (**Figure 1E**). Immunohistochemical staining showed positivity for desmin, MyoD1, and SMA (**Figures 1I–K**), initially suggestive of rhabdomyosarcoma. However, the diagnostic picture was complicated by the absence of typical alveolar or embryonal morphology and the unusual presentation in a 46-year-old male, prompting consideration of alternative diagnoses including dedifferentiated liposarcoma. According to the AJCC 8th edition staging system for soft tissue sarcoma, the tumor was classified as pT4N0M0, high-risk group, based on renal invasion. Ten days after the initial surgery, the patient developed a right scrotal mass. Contrast-enhanced CT of the pelvis showed a 5 cm exophytic tumor at the spermatic cord root with marginal enhancement (**Figure 1B**). Given the recent diagnosis of a high-grade retroperitoneal sarcoma, the differential diagnosis included either a second primary tumor or metastatic disease. The patient subsequently underwent radical orchiectomy under combined spinal-epidural anesthesia. Intraoperative findings revealed a normal-shaped right testis with a 5 cm cauliflower-like tumor at the spermatic cord root, adherent to the epididymis with tumor hemorrhage. The testis, epididymis, spermatic cord, and tumor were completely excised en bloc. After surgery, the patient received intravenous antibiotics (cefuroxime 1.5 g twice daily for three days) and analgesics as needed, with no complications, and was discharged on postoperative day 3. H&E staining of the testicular mass showed similar morphology to the retroperitoneal tumor (**Figures 1F–H**), and immunohistochemistry confirmed Desmin positivity (**Figure 1L**). Given the diagnostic uncertainty and the unusual presentation of two synchronous sarcomas at different anatomic sites, both specimens were referred for expert pathological consultation with fluorescence *in situ* hybridization (FISH) analysis. The final diagnosis was dedifferentiated liposarcoma with prominent heterologous myogenic differentiation, confirmed by demonstration of MDM2 and CDK4 gene amplification (cluster pattern) in both tumors (**Figures 1M, N**), along with consistent immunohistochemical expression of MDM2, CDK4, desmin, myogenin, and MyoD1. The Ki-67 proliferation index was 70-80% in both tumors, indicating highly aggressive tumor biology. Of note, the initial immunohistochemistry performed at the primary institution had failed to detect MDM2 and CDK4 expression, highlighting the critical importance of molecular testing in resolving this diagnostic dilemma. Postoperative chest CT performed three weeks after the initial surgery revealed multiple new solid pulmonary nodules, the largest measuring 20 mm, consistent with metastatic disease (**Figure 1C**). The patient declined adjuvant therapy after shared decision-making and was alive with metastatic disease at the four-week follow-up. This case underscores the aggressive nature of this rare tumor.

## Discussion

The main strength of this case report is its detailed documentation of an extremely rare clinical event—retroperitoneal dedifferentiated liposarcoma (DDLPS) with heterologous myogenic differentiation that metastasized to the testis within ten days after surgery and to the lungs within three weeks—with the diagnostic dilemma resolved by fluorescence *in situ* hybridization (FISH). However, this study has several limitations. First, as a single case report, the findings may not be generalizable. Second, the patient did not receive adjuvant therapy due to his postoperative recovery status and personal preference, so the impact of systemic treatment on this rare variant could not be evaluated. Third, FISH analysis was performed at a referral institution rather than the primary hospital, reflecting real-world diagnostic challenges in resource-limited settings. Long-term follow-up data are still being collected. The diagnostic challenge in this case arose from the tumor’s expression of myogenic markers (desmin, MyoD1, SMA), which initially suggested rhabdomyosarcoma (RMS) (1, 2). However, RMS is extremely rare in adults, and the patient’s age of 46 years was atypical. The definitive diagnosis of DDLPS with heterologous myogenic differentiation was established by FISH, which demonstrated MDM2 and CDK4 gene amplification in both tumors (3). Notably, MDM2/CDK4 immunohistochemistry (IHC) was negative at the primary institution but positive by FISH. This discrepancy between negative IHC and positive FISH in our case can be explained by three potential mechanisms. First, the primary institution may have used IHC antibodies with lower sensitivity or suboptimal antigen retrieval protocols. MDM2 and CDK4 protein expression levels can vary depending on fixation time, antibody clone, and detection system, and not all commercial antibodies perform equally well on routine surgical specimens. Second, low-level gene amplification below the IHC detection threshold can still be reliably detected by FISH. Studies have shown that FISH can detect as few as four to six copies of the MDM2 gene, whereas IHC requires higher protein expression levels to produce a visible signal. Third, heterogeneous amplification within the tumor may have resulted in sampling bias—the tissue section sent for IHC may have come from an area without amplification, while the FISH analysis was performed on a different area that contained amplified cells. Gambella et al. systematically evaluated MDM2 FISH patterns in liposarcoma and identified three amplification patterns (scattered, clustered, and mixed), noting that heterogeneous distribution of amplified signals can lead to false-negative results if only limited tumor areas are analyzed. Their study provides practical troubleshooting recommendations for such discordant IHC-FISH cases (12).Aslam et al. reported that while CDK4 IHC has a sensitivity of 82.6% for detecting DDLPS, FISH remains the confirmatory gold standard, particularly when IHC results are negative (4). Thway et al. further demonstrated that FISH for MDM2 amplification has a high correlation rate with histology and is particularly useful in histologically equivocal cases, such as DDLPS without a corresponding well-differentiated component (5).A recent multidisciplinary position statement by Haddox et al. emphasizes that expert pathology review and appropriate application of diagnostic molecular techniques, including MDM2/CDK4 FISH, are key components of DDLPS diagnosis (11). This case underscores a critical lesson for pathologists and clinicians: negative MDM2/CDK4 IHC does not exclude the diagnosis of DDLPS, especially when heterologous differentiation is present. FISH should be performed whenever clinical or morphological suspicion remains high, regardless of IHC results. A systematic review of the literature reveals fewer than ten reported cases of DDLPS with rhabdomyoblastic differentiation (1, 2, 6, 7). Ichikawa et al. recently reviewed the clinical features of heterologous differentiation in DDLPS, noting that approximately 5-10% of DDLPS show heterologous differentiation, with myogenic being the most common subtype (10).Our case is unique in three respects: it demonstrates the shortest interval to metastasis reported to date (ten days to testis, three weeks to lung); it is the first to document testicular metastasis from this tumor type; testicular metastases from sarcomas are exceptionally rare, representing less than 1% of all testicular malignancies (14);and it highlights the diagnostic pitfall of IHC-negative but FISH-positive MDM2/CDK4 status. The presence of venous tumor thrombi in the testicular specimen supports hematogenous dissemination as the mechanism of rapid spread, consistent with the patterns of recurrence described by Gronchi et al., who reported that liposarcoma histology influences the pattern of failure and that distant metastases occur in a subset of aggressive cases (8). The patient was discussed in the institutional multidisciplinary tumor board. CDK4 inhibitors (e.g., palbociclib) represent a potential targeted therapy for MDM2/CDK4-amplified DDLPS, with a phase II trial reporting a progression-free survival rate of 66% at twelve weeks (9). Long-term overall survival data from the palbociclib phase 2 trial recently reported a median OS of 25.6 months, further supporting the role of CDK4/6 inhibition in this disease (13).However, patients with heterologous differentiation were excluded from that trial, highlighting a knowledge gap. The patient ultimately declined adjuvant therapy after shared decision-making. In conclusion, this case provides three key lessons for clinicians. First, any retroperitoneal sarcoma in an adult expressing myogenic markers should undergo MDM2/CDK4 FISH testing, regardless of initial IHC results. Second, aggressive DDLPS variants with heterologous differentiation can metastasize rapidly, suggesting that early postoperative surveillance with chest CT and scrotal ultrasound should be considered within two to four weeks. Third, patients with this rare entity should be referred to a multidisciplinary sarcoma center for individualized treatment planning, and future clinical trials of targeted therapies should explicitly include this high-risk subgroup.

## Patient perspective

The patient provided informed consent for publication of this case report. He expressed gratitude for the timely diagnosis and surgical treatment. He was initially confused by the rapid development of a scrotal mass shortly after the major abdominal surgery, which caused significant anxiety. After the final diagnosis was established and the treatment plan was explained, he gained a clearer understanding of his condition. He agreed to share his clinical data to help other patients with similar rare conditions.

### Informed consent

Written informed consent was obtained from the patient for the publication of this case report and any accompanying images. A copy of the written consent is available for review by the Editor-in-Chief of this journal.

### Limitations

This study has several limitations. First, as a single case report, the findings may not be generalizable to all patients with dedifferentiated liposarcoma with heterologous myogenic differentiation. Second, the patient did not receive adjuvant chemotherapy or radiotherapy following surgical resection due to his postoperative recovery status and personal preference; therefore, the impact of systemic therapy on this rare tumor variant could not be evaluated. Third, long-term follow-up data are still being collected, and the patient’s ultimate survival outcome remains to be determined.

## Conclusion

This case report describes a rare presentation of dedifferentiated liposarcoma (DDLPS) with heterologous myogenic differentiation arising in the retroperitoneum with subsequent testicular metastasis in a 46-year-old male. The diagnostic journey illustrates several critical lessons. First, DDLPS with rhabdomyoblastic differentiation represents a significant diagnostic pitfall, as expression of myogenic markers (desmin, myogenin, MyoD1) can easily lead to misclassification as primary rhabdomyosarcoma, a distinction of paramount clinical importance given their substantially different treatment paradigms. Second, the demonstration of identical *MDM2* and *CDK4* gene amplification in both the retroperitoneal and testicular specimens via FISH analysis confirmed a single disease process and highlights the critical role of molecular testing in resolving diagnostic dilemmas. Third, the rapid postoperative development of testicular and pulmonary metastases underscores the aggressive biological behavior of this rare tumor variant. For any retroperitoneal or paratesticular sarcoma expressing myogenic markers, particularly in adult patients, *MDM2*/*CDK4* FISH testing should be considered an essential component of the diagnostic workup to avoid misdiagnosis and ensure appropriate therapeutic planning.

## Data Availability

The original contributions presented in the study are included in this article. Further inquiries can be directed to the corresponding author.
